# Deciphering microbiota-host interplay: bacterial metabolites and protein post-translational modifications

**DOI:** 10.1093/lifemedi/lnae003

**Published:** 2024-02-06

**Authors:** Mingya Zhang, Sangkyu Lee, Min Huang, Minjia Tan

**Affiliations:** State Key Laboratory of Drug Research, Shanghai Institute of Materia Medica, Chinese Academy of Sciences, Shanghai 201203, China; School of Pharmacy, Sungkyunkwan University, Suwon 16419, South Korea; State Key Laboratory of Drug Research, Shanghai Institute of Materia Medica, Chinese Academy of Sciences, Shanghai 201203, China; State Key Laboratory of Drug Research, Shanghai Institute of Materia Medica, Chinese Academy of Sciences, Shanghai 201203, China

The critical role of the gut microbial community in host physiology and pathology has been increasingly appreciated. The symbiotic relationship between gut microbiota and host maintains the host’s health or triggers pathological alterations. While the mechanistic understanding of this interaction remains very limited, it has been widely accepted that microbial metabolites are important mediators. Gut microbial metabolites can potentially interact with various types of host cells, such as colonic mucosa cells that are constantly exposed to the environment of bacterial metabolites, and affect cellular functions. Bacterial metabolites could function as signal molecules or metabolic substrates, triggering signal transduction, metabolic rewiring, and epigenetic or transcriptional regulation, which fundamentally contribute to cellular functions. Finally, it has emerged that metabolites could affect protein post-translational modifications (PTMs), suggesting a new possible linkage between gut microbial metabolites and host cells. Current understanding of host PTMs influenced by gut microbial metabolites is by far limited. A major hurdle toward this goal is a lack of efficient method for the discovery of low stoichiometry PTMs even using state-of-the-art mass spectrometry (MS)-based proteomics technology. At present, the microbial metabolite-regulated host PTMs can be largely divided into two categories: one is the direct covalent modifications of host proteins from microbial metabolites; the other is the regulation of PTMs by enhancing or suppressing the corresponding regulatory factors.

**Figure 1.**
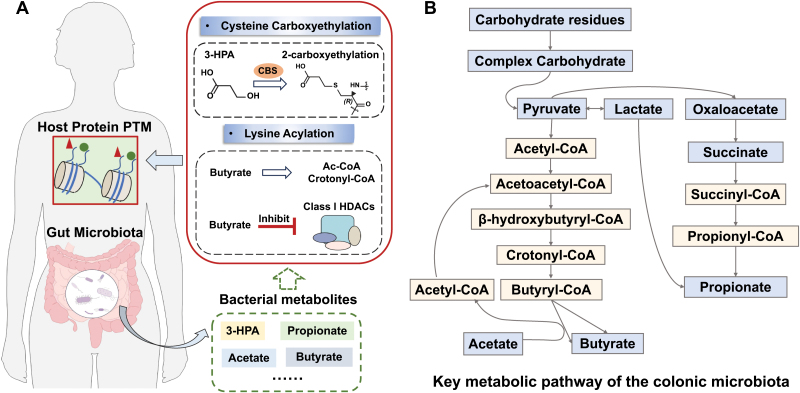
**The role of microbial metabolites in host PTM regulation.** (A) PTMs are mediated by gut microbial metabolites occurring in host cells. (B) Metabolic pathways of the colonic microbiota in the process of fermentation. 3-HPA, 3-hydroxypropionic acid; CBS, cystathionine β-synthase; SCFAs, short-chain fatty acids.

Using high-resolution MS-based technology, a recent study published in *Science* revealed a novel microbial metabolite-derived PTM, named cysteine carboxyethylation, which is associated with human leukocyte antigen (HLA)-restricted autoimmunity [1]. In this study, to explore the possibility of PTM as a source of neonatal antigens *in vivo*, the authors applied an open search algorithm for MS data analysis to un-restrictively identify all possible mass shifts (delta mass) of protein canonical amino acid residues in peripheral blood mononuclear cells (PBMCs) from ankylosing spondylitis (AS) patients. These mass shifts of amino acid residues likely correspond to potential PTMs, which are different from the translated products of genome coding sequences. In such analysis, 643 unique delta mass clusters were identified in total. Interestingly, an amino acid derivative with a 72.021 Da mass shift was significantly increased in AS patients, especially in the integrin aIIb (ITGA2B). This mass shift was commonly detected at Cys, Arg, and Trp residues, among which Cys + 72.021 was the most dominant. Cys + 72.021 fits best to a normal distribution compared with frequency distributions of other amino acid residues + 72.02. And the molecular formula of this mass shift matched best to C_3_H_4_O_2_. According to the elemental composition and potential products of a condensation reaction, this modification was deduced to be the chemical structure of a hydroxypropionyl group or a carboxyethyl group. Further chemical and biochemical experiments demonstrated that this modification is carboxyethylation of cysteine with a thioether bond to 3-hydroxypropionic acid (3-HPA). To identify the regulatory enzyme of cysteine carboxyethylation, the authors conducted co-immunoprecipitation (coIP)-MS experiment using anti-ITGA2B antibody in HEK293T cell lysates. The results showed the carboxyethylated ITGA2B Cys96 (ITGA2B-ceC96) was associated with the l-cysteine metabolic process, which is linked to cystathionine β-synthase (CBS). As previous studies showed that CBS could convert serine (similar structure with 3-HPA) and homocysteine (similar structure with cysteine) to cystathionine in the folate pathway, CBS was presumed to be a potential enzyme that promotes cysteine 2-carboxyethylation formation from cysteine and 3-HPA (Fig. 1A).

Next, the authors assayed plasma samples from AS patients and healthy donors and found that AS group showed higher levels of autoantibodies against ITFA2B-ceC96. Subsequently, they showed the ITFA2B-ceC96 peptides promoted an antigen-specific T-cell response that was restricted to HLA-DRB1*04. Cys96 carboxyethylation can potentially influence the binding of HLA class II molecules and alter the surface available for T cell receptor (TCR) recognition. It was further verified that carboxyethylated ITGA2B was associated with autoantibodies and T-cell responses in HLA-DRB1*04 in AS patients. They finally demonstrated that ITFA2B-ceC96 immunization could lead to increased susceptibility to spondylitis in the immunized HLA-DR4 mice model. Together, this study revealed a new diagram of human–environment interactions in autoimmune diseases, in which bacterial metabolite-derived PTM plays an essential role in generating neoantigen. This study also raised some interesting questions that require further investigation. For example, what is the biological origin of 3-HPA in human? How are 3-HPA levels elevated in patients with AS? Are there other cysteine carboxyethylation substrates in cells? What other cellular functions that cysteine carboxyethylation could have?

Coenzyme A adducts and corresponding short-chain fatty acids (SCFAs) serve as important donors for lysine acylations, which intimately link cellular metabolism to epigenetic regulation [2]. Currently, these lysine acylation donors are generally considered to be derived from host cells. Indeed, almost all these CoA donors can be also generated by a microbe. For example, in the colonic microbiota, SCFA-CoAs can be generated from several pathways, such as CoA-transfer pathway, succinate pathway, and butyrate kinase pathway, in the process of fermentation (Fig. 1B). The carbohydrate residues are converted to complex carbohydrates. Next, the intermediate metabolites derived from complex carbohydrates are further converted to CoAs, such as acetyl-CoA, proionyl-CoA, butyryl-CoA, and crotonyl-CoA [3]. It remains unclear whether these bacteria-derived CoAs can also be utilized for host lysine acylations.

In addition to serving as PTM donor, microbial metabolites can directly regulate the activity of PTM regulatory enzymes. For example, some SCFAs produced by intestinal bacteria through digestion and fermentation of dietary fiber, such as acetate, propionate, and butyrate, are reported to regulate the lysine deacylase activity. Patrick et al. [4] demonstrated that butyrate promotes histone crotonylation in the colon through inhibiting histone deacetylase activity. In this study, by using a pan-crotonylation antibody and high-performance liquid chromatography–tandem mass spectrometry analysis, they found that histone lysine crotonylation was abundant in the crypts of the small intestine and colon. In a mouse model fed with a cocktail of antibiotics for 3 days, the authors observed the decrease of gut microbiota that was positively correlated with the decrease of histone crotonylation and the increase of histone deacetylases (HDACs) in the mouse large intestine. Furthermore, *in vitro* assays of recombinant showed class I histone deacetylase could serve as decrotonylase. Interestingly, expressions of HDAC2 were increased in the antibiotic cocktail-treated mouse model. Immunoblotting analysis using a pan-crotonylation antibody showed the exogenous crotonate and butyrate elevated histone crotonylation levels on H3 and H4 in both cells and mouse small intestine. *In vitro* incubation experiment showed HDACs could regulate histone crotonylation. In contrast, treatment of HDAC inhibitors (butyrate and trichostatin-A (TSA)) could increase histone crotonylation (Fig. 1A). These results suggested that SCFAs, such as butyrate, could promote histone crotonylation due to the inhibition of HDAC activities in colon cells. Thus, this study shows gut microbiota regulates HDACs by producing short-chain fatty acids, which impacts intestinal lysine crotonylation, and in turn, regulates gene expression and cellular functions.

As gut microbes can produce myriad types of metabolites, it could be postulated that some of these compounds, especially those high-energy intermediates, could serve as donors or indirectly impact the activity of PTM enzymes to regulate host protein PTMs, which could further impact the cellular functions of the host. Since these postulated bacteria-derived PTMs are likely to be in extremely low abundance with highly diverse chemical structures, new technology development for the improvement of MS detection sensitivity and new computational algorithm for more efficient identification of unknown PTMs would be expected to expedite the discovery process.
